# Supplementary material for: Effectiveness of app-based cognitive behavioral therapy for insomnia on preventing major depressive disorder in youth with insomnia and subclinical depression: A randomized clinical trial
Source: PLoS Med. 2025 Jan 21;22(1):e1004510. doi: 10.1371/journal.pmed.1004510 (PMC11750088; doi:10.1371/journal.pmed.1004510)
Supplement: S1 Protocol — (PDF) [file pmed.1004510.s001.pdf]

Effectiveness of e-based cognitive behavioral therapy for insomnia on improving mental health in Chinese youths with insomnia: a large-scale randomized control trial

**Principal Investigators**	Yun Kwok Wing, FRCPsych, Chinese University of Hong Kong; Lin Lu, PhD, Peking University Sixth Hospital
**Co-Investigators**	Ji-Hui Zhang, PhD, Chinese University of Hong Kong; Ngan Yin Chan, PhD, Chinese University of Hong Kong; Le Shi PhD, Peking University Sixth Hospital; Shirley Xin Li, PhD, Hong Kong University

Introduction

Depression in Youth

Youth is a sensitive and critical period of development marked by considerable changes in biological, cognitive, psychological and social functioning. It is also a vulnerable transitional stage often linked to the emergence of mental health problems. In particular, major depressive disorder (MDD) is among the most common psychiatric disorders in the youth population, and has become the leading cause of disability adjusted life-years (DALYs) for females aged 20-24 year.[1] Two large epidemiological surveys reported that depressive symptoms were present in 13.8%- 21.0% of college students, and a recent meta-analysis showed that the prevalence estimate of depression or depressive symptoms among medical students is 27.2%.[2-4] Youth depression is associated with substantial psychosocial and functional impairments (e.g. poor school achievement, impaired social skills, interpersonal difficulties, substance abuse) and an elevated risk of suicidal behaviour and completed suicide.[5, 6] In addition, youth depression has been found to predict recurrent depression in adulthood and be associated with long-term functional impairment.[7, 8] Although the treatments for youth depression, such as cognitive-behavioral therapy and interpersonal approaches, have been widely investigated with substantial progress, non-response to treatment, residual symptoms and relapses/recurrence were commonly reported (e.g. up to 50% of the treated adolescent cases experienced a recurrence).[9] Moreover, young people are often reluctant to initiate help seeking face-to-face for mental health problems, because of the fear of stigma and a preference of self-reliance.[10] Thus, youth depression is often left untreated. According to national surveys conducted in America, only 24% of college students with depression were under appropriate treatment.[11] In view of its high prevalence, persistence, deleterious long-term impacts and treatment challenges, there is an imperative need to develop and implement an effective prevention programme for depression, especially during early developmental stage, from a public health perspective.

Insomnia as a Target for Prevention of Depression – Rationale, Empirical Basis & Research Gaps

Interplay between insomnia and depression

Targeting insomnia may become a promising intervention to prevent depression among youth, as Insomnia and depression are highly comorbid conditions, with increasing evidence suggesting their intricate, bidirectional relationship. In particular, insomnia and depression often co-occur, with 35-47% of individuals with insomnia having clinically significant depression and 60-84% of people with MDD experiencing comorbid insomnia symptoms.[12] Rather than a symptom of depression, insomnia is an independent problem which commonly precedes the development of depression,[13] and has been found to increase the risk of developing incident new depression,[14, 15] lead to poorer treatment response,[16] increase the susceptibility of having recurrence,[17] and represent an independent risk factor for suicidal ideation and behaviour.[18-20] This growing body of evidence emphasise the unique, independent role of insomnia in the course of MDD, and interventions which directly target insomnia are most likely to improve depression outcomes, or may even prevent depression.

The mechanisms underlying the comorbidity of depression and insomnia have provided clues for further validating the preventive effect of insomnia treatments on depression. In

biological domain, the most widely adopted theory is hyperarousal theory.[21, 22] Evidence has clearly shown that stress-activated hypothalamic–pituitary–adrenal (HPA) axis hyperactivity exists in both depressed and insomniac patients. [23-25] Moreover, arousal responses of HPA axis is associated with slow wave sleep deficit and sleep continuity disruption in both depression and insomnia.[26-28] In psychological domain, maladaptive cognitions and behaviors, such as worry, ruminations, vulnerability to stress and poor problem solving skills, are implicated in the association between depression and insomnia.[22, 29] Additionally, a temporal relationship between depression and insomnia also favours the idea that effective treatment of insomnia would lower the risk of depression. As mentioned above, the occurrence of insomnia commonly precedes depression in most cases, and several longitudinal studies have demonstrated that previous insomnia was related to subsequent onset of depression.[30-33] A meta-analytic review revealed that insomniac patients without depression at baseline have a twofold risk to develop depression.[15] These evidences further indicated that insomnia could play a causal role in the onset of depression.[26]

Cognitive behavioral therapy for insomnia

Cognitive behavioral therapy for insomnia (CBT-I) is a multi-component, non-pharmacologic intervention that targets behavioral, cognitive and physiological perpetuating factors of insomnia. There is a strong evidence base to support the short-term and long-term efficacy of CBT-I for chronic primary insomnia in adults.[34, 35] A meta-analysis has validated the comparable treatment efficacy between CBT-I and pharmacotherapy during short-term treatment,[36] and CBT-I is more effective than pharmacotherapy in long-term treatment.[37] Additionally, CBT-I can benefit youth other than adults. A recent systematic review showed that CBT-I had large effects on various sleep variables and medium effects on mental health among college students, whereas other psychological interventions such as sleep hygiene and relaxation showed small to medium effects for sleep variables.[38] Moreover, the preliminary results from our ongoing research showed that CBT-I significantly improved insomnia symptoms in adolescent patients with insomnia (Trial registration: clinicaltrials.gov Identifier: NCT03522701) and could prevent incident acute insomnia in the high-risk adolescents who genetically predisposed to insomnia and presented with subclinical insomnia symptoms (Funded by General Research Fund, Ref. CUHK 4012-PPR-11).

Apart from its direct effects on sleep improvements, CBT-I can also be effective in reducing depressive severity. In line with the findings in previous studies [36, 61-63], our research team has validated the effectiveness of CBT-I for improving both depressive and insomnia symptoms in patients suffering insomnia and depression (Funded by Health and Medical Research Fund, Ref. 10110341). In a randomized trial which compared treatment effects of CBT-I and CBT for depression (CBT-D) in patients with comorbid MDD and insomnia, CBT-I was more effective than CBT-D in reducing insomnia severity, and equally effective in reducing depressive severity [64], indicating the importance of applying insomnia treatment for patients with both insomnia and depression.

Digital approach to treatment of insomnia for preventing depression in youth

While face-to-face CBT-I has shown promise in management of insomnia and psychiatric conditions such as depression, several barriers to implementation remain in clinical practice. Firstly, face-to-face CBT-I appears to be time-consuming for therapists in practice.[39] Moreover, there are not enough therapists qualify for offering CBT-I.[40, 41] Thus, it is important to develop alternative delivery modalities of CBT-I which have comparable efficacy of standard face-to-face CBT-I and can improve its feasibility. Two major variants of CBT-I that may address barriers impacting CBT delivery are internet-delivered CBT-I (e-CBT-I) and group CBT-I. A recent meta-analysis revealed that e-CBT-I and face-to-face CBT-I had comparable effects on insomnia and other sleep-related symptoms,[42] and meta-analysis of group CBT-I showed that group CBT-I is also an effective treatment for insomnia, as it has large effect sizes for sleep onset latency, sleep efficiency, and wake after sleep onset.[43] As for youth with insomnia, a series of studies conducted by a research team in University of Amsterdam showed that both e-CBT-I and group CBT-I were well accepted and highly feasible with low dropout rates.[44, 45] Further, two treatment modalities were effective for youth with insomnia and no significant differences were found between these two formats. However, this research team found that e-CBT-I was more cost effective than group CBT-I in treating youth insomnia.[46] Moreover, internet has the capacity to reach a large group of young people given their increased accessibility and frequent use of web and smartphone technology,[47, 48] and is important in their daily lives.[49] Therefore, e-CBT-I may be a preferable alternative for youth with insomnia.

Currently, researchers have done considerable work to investigate whether e-CBT-I could be effective in improving psychological well-being for adults. To our knowledge, to date there is two randomised controlled trial (RCT) to particularly test whether indicated preventive intervention for insomnia could prevent depression in adults.[50, 51] Christensen et al. and Cheng et al. both showed promising results of e-CBT-I in alleviating depressive symptoms in their study, and the effects persisted at 1-year follow-up and 18-month follow-up, respectively. However, Christensen et al. found that emergence of MDD was low in targeted sample and did not conduct clinical assessment at 18-month follow-up. By using self-report scale instead of clinical assessment, Cheng et al. found that in those with minimal to no depression at baseline, the incidence of moderate to severe depression at 1-year follow-up in the dCBT-I condition was less than half that in the control condition. Another study further demonstrated that the effects of e-CBT-I on improving psychological health were mediated by a reduction of insomnia symptoms.[52]

Despite these findings, there are some issues of current studies that need to be addressed in the future. As the existing data on e-CBT-I mostly came from the studies based on the adult samples, little is known as to whether timely intervention of sleep disturbance could potentially serve as a preventive measure to alter the trajectory of youth depression. Nevertheless, previous studies showed that improving sleep via e-CBT-I in college students leaded to a reduction in psychotic symptoms such as hallucinations and paranoia,[53] and e-CBT-I could also alleviate anxiety severity in college students with insomnia.[54] Therefore, further research is warranted to evaluate the effects of e-CBT-I on reducing depressive symptoms in youth. Another issue is that it is still unclear by which mechanisms e-CBT-I might affect mental health. The improvement of mental health

achieved with e-CBT-I might simply attribute to the reduction of insomnia symptoms as indicated in previous study,[52] or it might due to reverse the effects of poor sleep hygiene, sleep-related unhelpful thoughts and maladaptive behaviors, circadian rhythm disruption and chronic sleep deprivation.[54] Additionally, previous studies mainly focused on evaluating the changes of psychological outcomes, and only one study showed that e-CBT-I could lower the risk of depression. In this study, depression was assessed by self-report scale instead of clinical assessment. Thus, the findings should be replicated and assessments that are more reliable should be adopted in other independent studies.

To address the limitations in the existing literature, we propose to conduct a RCT of targeted prevention intervention to validate the treatment effect of e-CBT-I on insomnia disorder and examine whether digitally delivered insomnia treatment can reduce the risk of depression in a high-risk group of youth population. To further explore the mechanisms underlying the association between insomnia treatment and depression, we will evaluate whether changes in candidate factors including insomnia symptoms, mood symptoms, sleep-related unhelpful thoughts and maladaptive behaviours, and circadian rhythm disruption will mediate the changes in depressive symptoms. On a conceptual level, the proposed interventional study will also allow us to further test the causal pathway between sleep disturbance and major depression, by intervening and treating insomnia as a preventive measure of depression. On a pragmatic level, digital approach to insomnia intervention for prevention of youth depression, if effective, may have important public health implications given its high accessibility to the young populations.

Methodology

Study Design

A randomized, assessor-blind, parallel group-controlled trial will be conducted in youth with insomnia and subclinical depression. All qualified participants will be randomly assigned via online system to receive e-CBT-I or health education (HE). Randomization will be stratified by sex and the severity of insomnia. Assessments will be carried out at baseline, post-treatment, 6-month follow-up and 12-month follow-up. Additional assessments will be given upon the completion of sessions 2 (post-session 2) and 4 (post-session 4) to evaluate sleep symptoms, mood symptoms, circadian rhythms and sleep parameters of participants. Saliva collection kit will be mailed to participants at baseline to collect their salivary cortisol and DNA, and at 12-month follow-up to collect their salivary DNA. Semi-structural individual interviews about user experience and satisfaction towards e-CBT-I intervention via phone calls will be given to part of the participants in e-CBT-I group after intervention.

Main hypothesis in the present study include:

1. Youth receiving e-CBT-I will have fewer onsets of depressive disorder and higher rates of remission in insomnia disorder at post-intervention, 6-month and 12-month compared to those in control group.
2. Youth in e-CBT-I group will have fewer insomnia symptoms, depressive symptoms, suicidal ideation, plans and attempts than those in control group at each timepoint.
3. Changes in candidate factors including insomnia symptoms, mood symptoms, circadian rhythms and sleep parameters will mediate the outcomes.

Recruitment and Screening

Potential participants will be recruited from colleges and senior secondary school in Hong Kong and mainland China and will be identified from various sources. These will include the local partner schools in Hong Kong established from our previous research projects. Partner schools in mainland China will be recruited by our collaborators with the school links in mainland China. Through our partner schools, students will be introduced to this study, and they will be asked to return the signed informed consents and leave their contact (e.g., email) in the reply slip to the research team if they are interested in taking part in the study. Furthermore, potential participants will be recruited through community referrals and advertising via social media and emails in Hong Kong and mainland China. Interested participants will be asked to complete the screening assessments, including Patient Health Questionnaire-9 (PHQ-9) [55] and Insomnia Severity Index (ISI) [56] via an online portal (Qualtrics). Participants who have an ISI score of no less than 15 and a PHQ-9 score between 4 and 20 will be further invited for diagnostic interview through telephone to screen for insomnia (International Statistical Classification of Diseases and Related Health Problems10th Revision Classification of Mental and Behavioral Disorders, ICD-10 Classification of Mental and Behavioral Disorders [57]), MDD and suicidality including ideation, plans and attempts (Mini International Neuropsychiatric Interview, MINI [58]). A potential participant who meets the inclusion criteria (see below) will be eligible to take part in the study.

Participants

Inclusion criteria and exclusion criteria for participants are as follows:

Inclusion criteria

1. Native Han youth in Hong Kong and mainland China, aged between15-25
2. A diagnosis of insomnia disorder measured by ICD-10 Classification of Mental and Behavioral Disorders
3. The presence of moderate or severe insomnia measured by a score of 15 or above on ISI
4. The presence of subclinical depression assessed by a score of more than 4 but less than 20 on PHQ-9
5. Access to smartphones
6. Ability to read and understand research protocol

Exclusion criteria

1. Shift workers
2. The presence of prominent suicidality (suicide plans and suicide attempts) measured by MINI via telephone interview
3. A clinical diagnosis of psychosis, schizophrenia, bipolar disorder, or neurodevelopmental disorders
4. Current medical conditions that could cause poor sleep quality and sleep continuity disruption, such as eczema
5. The presence of current MDD or a prior episode of MDD within past two months measured by MINI via telephone interview
6. Women during pregnancy or lactation

7. Currently receiving psychological treatment for insomnia provided by a psychologist and/or pharmacological treatment for depression

Intervention

The e-CBT-I will be delivered by a mobile application (eSleep) which contains a digital, self-paced, and highly interactive programme. The Chinese version (simplified Chinese/Mandarin) of the application has been designed to adapt for the language background of the participants in China. The program is developed as based on well-established CBT-I treatment protocol and consists of six 20-30 minutes sequential modules unlocked weekly with animated elements, including an overview of sleep, sleep restriction, stimulus control, cognitive therapy, structured worry time and relapse prevention. During sleep restriction module (session 2), participants will obtain a "sleep prescription" from the application, specifying their sleep window (from initial bedtime to final arising time) for the next seven days. The setting of the sleep window is based on individual's average total sleep time from the previous week, while adhering to the 5-hour minimum rule. The sleep prescription is adjusted weekly based on the following criteria: 1) if sleep efficiency > 85%, increase sleep window by 15-20 min; 2) if sleep efficiency < 80%, decrease sleep window by 15-20 min; and 3) if sleep efficiency is between 80%-85%, keep sleep window the same. The application control management system provides online analytics, which allows the researchers to monitor individual's participation during the intervention by assessing how many sessions were completed and the number of weeks to complete the course. Participants will have access to the e-CBT-I treatment for 12 weeks, allowing them additional time to complete the intervention in case they were unable to completed it within the initial 6-week period. Individualized text messages will be sent to the participants regularly, asking them to complete the treatment sessions and sleep diaries on time. The participants will also be contacted weekly to prevent possible dropout from occurring during the intervention. The PI and collaborators have had the research and clinical experiences in delivering CBT-I, and the e-CBT-I treatment protocol has been tested and modified from our previous studies [Trial registration: clinicaltrials.gov Identifier: NCT03438331; funded by Early Career Scheme, Ref. 27613017] [59].

The HE, a psychoeducation/information-approach, delivered in control group has been adopted in the previous study [50]. It also consists of six consecutive sessions which contains information about general sleep knowledge, functions of human organs, nutrition, environmental health, brain health, identification and treatments of common diseases, but the contents are not related to any active therapeutic components of CBT-I. Each module was unlocked weekly, and participants will have access to the intervention for 12 weeks. The general sleep knowledge session was purposively added in this sleep related RCT to meet the expectation of the participants. Individualized text messages will be sent to the participants regularly to remind them of completing the sessions and sleep diaries on time. The participants will also be contacted weekly to prevent possible dropout from occurring during the intervention.

Assessments

Assessments will be delivered at baseline, post-treatment, 6-month follow-up and 12-

month follow-up. At baseline, all participants will be interviewed for their basic demographic and clinical characteristics (age, sex, education, height, weight, current medications etc). Self-administered questionnaires will be completed online to assess depressive symptoms and severity (PHQ-9), anxiety (Generalised Anxiety Disorder 7-item, GAD-7 [60]), suicidal ideation (Beck Scale for Suicide Ideation, BSSI [61]), sleep symptoms (ISI, 7-Day Daily Sleep Diary), daytime symptoms (Multidimensional Fatigue Inventory, MFI [62]), chronotype (reduced Morningness-Eveningness Questionnaire, rMEQ [63]), sleep-related thoughts and behaviors (brief version of dysfunctional beliefs and attitudes about sleep, DBAS-16 [64]), circadian rhythms and sleep parameters (7-Day Daily Sleep Diary). During the intervention, participants will additionally complete ongoing assessments to evaluate their sleep symptoms, mood symptoms and sleep parameters (ISI, PHQ-9, GAD-7, 7-Day Daily Sleep Diary) at post-session 2 and post-session 4. Saliva collection kit will be mailed to participants at baseline to collect their salivary cortisol and DNA, and at 12-month follow-up to collect their salivary DNA. After finishing the intervention, each participant will be paid CNY100 (or HKD equivalent) for his or her participation. Part of the participants in e-CBT-I group will be invited to receive semi-structural individual interviews about user experience and satisfaction towards e-CBT-I intervention via phone after intervention, and will be additionally paid CNY100 (or HKD equivalent) for his or her participation.

At post-treatment, 6-month follow-up and 12-month follow-up, participants will receive phone calls from the independent researcher blinded to the randomization status for diagnostic interviews to examine whether they have current insomnia measured by ICD-10 Classification of Mental and Behavioral Disorders, MDD or suicidality measured by MINI. Part of the diagnostic interviews will be audiotaped and second-rated by an independent rater to assess interrater reliability after obtaining participants' consents. In addition, participants will complete online questionnaires similar to those at baseline. After finishing the 12-month follow-up, each participant will be paid CNY50 (or HKD equivalent) for his or her participation.

Outcomes

The primary outcomes include remission rate of insomnia disorder conformed by ICD-10 Classification of Mental and Behavioral Disorders, change of insomnia symptoms measured by ISI, occurrence of MDD conformed by MINI and change of depressive symptoms measured by PHQ-9. Secondary outcomes include incidence of suicidality which includes plans and attempts as measured by MINI and change of anxiety symptoms measured by GAD-7. Other pre-specified outcome include incidence of suicidal ideation measured by BSSI, change of daytime symptoms measured by MFI, change of sleep-related thoughts and behaviors measured by DBAS-16, change of circadian rhythms measured by 7-Day Daily Sleep Diary and MEQ, and change of sleep parameters including time in bed (TIB), total sleep time (TST), sleep onset latency (SOL), wake after sleep onset (WASO), sleep efficiency (SE) measured by 7-Day Daily Sleep Diary.

Sample Size Estimation

The sample size calculation was based on the occurrence of diagnosed depressive

disorder because the primary aim of the current study was to prevent clinical depression. According to the previous meta-analysis, the incidence rate of depression is approximately 11% in the individuals with insomnia [15]. As the study sample included a mixture of individuals without prior depression and those with a history of MDD, an expected 50% increase in the cumulative one-year occurrence of diagnosed depressive disorder is anticipated (16.5%). Based on the previous research on CBT-I and our trial data on sleep-focused treatment for depression, a 50% reduction of occurrence rate is expected for the intervention group. Power analysis using Stata suggested that a total of 282 participants per condition would be required to achieve a power of 0.80 whilst allowing a type I error of 0.05 detect a hazard ratio of 0.5 under 1:1 randomization. In view of the attrition rate from previous study (40%), a total of 940 participants would be required (470 participants in each group x 2 groups). A protocol amendment was submitted to reduce the sample size to 708 (354 per group) based on an estimated attrition rate of approximately 20%, as it was determined that the attrition rate was lower than initially expected.

Data Analysis

All main analyses will be based on the intent-to-treat (ITT) approach. We aim to obtain full follow-up data on every participant, but missing data may be inevitably expected due to withdrawal, loss-to-follow-up, or non-response questionnaire items. Baseline characteristics will be presented as percentage for categorical variables and as means (standard deviations) for continuous variables. The primary and secondary outcomes with continuous variables will be examined by using linear mixed-effects model (LMM) analysis. The changes of efficacy indicators (e.g., PHQ-9) from baseline to each follow-up time points and intervention differences were tested by the MIXED module of Stata, with major measures at each time point nested within each participant. Interaction effects of group by time will be examined to compare the effects of treatment on the major outcomes. Between-group difference in the estimated mean change from baseline to each time points from mixed models and Cohen's *d* will be used to calculate effect sizes. Current MDD will be analyzed by the survival analysis and the Mentel-Cog log rank test. An observation will be right censored to the day after the last point of interview when a participant is no longer eligible to experience a depressive episode, e.g., lost to follow-up or completed the trial without experiencing a depressive episode (12-month follow-up after intervention). The effect of the intervention on insomnia remission will be analyzed using generalized estimating equations (GEE). The GEE analysis will be conducted by using the XTGEE module of Stata. Mediation analysis using maximum likelihood estimation within a structural equation modeling framework will be used to determine which outcomes are mediated by changes in candidate factors including sleep symptoms, mood symptoms, sleep-related unhelpful thoughts and maladaptive behaviors, circadian rhythm disruption and sleep parameters at follow-up. Sensitivity analyses will be conducted using imputation methods to examine the robustness of the results based on different assumptions about the missing data. Strata variables (sex and insomnia severity) at baseline will be included as covariates in all analyses. Variables with a p value ≤ 0.05 will be considered statistically significant.

Ethics & Other Considerations

All participants will provide their online informed consent before participation. Potential adverse effects will be evaluated by self-reporting of existing adverse effects for frequency and severity [65]. Participants will be excluded from the study and provided with information for further treatment if they are diagnosed with MDD or show a suicidal risk upon assessments. The proposed study will be conducted in compliance with the protocol, the ICH-CGP Guideline, and all other applicable regulatory requirements. The ICH shall include the elements required by the International Conference on Harmonization (ICH) Good Clinical Practice (GCP) Guideline and local regulation requirements, and will adhere to the ethical principles based on the Declaration of Helsinki.

References:

1. Mokdad AH, Forouzanfar MH, Daoud F, Mokdad AA, El Bcheraoui C, Moradi-Lakeh M, et al. Global burden of diseases, injuries, and risk factors for young people's health during 1990-2013: a systematic analysis for the Global Burden of Disease Study 2013. Lancet. 2016;387(10036):2383-2401.
2. Eisenberg D, Gollust SE, Golberstein E, Hefner JL. Prevalence and correlates of depression, anxiety, and suicidality among university students. Am J Orthopsychiatry. 2007;77(4):534-542.
3. Rotenstein LS, Ramos MA, Torre M, Segal JB, Peluso MJ, Guille C, et al. Prevalence of Depression, Depressive Symptoms, and Suicidal Ideation Among Medical Students: A Systematic Review and Meta-Analysis. JAMA. 2016;316(21):2214-2236.
4. Steptoe A, Tsuda A, Tanaka Y, Wardle J. Depressive symptoms, socio-economic background, sense of control, and cultural factors in university students from 23 countries. Int J Behav Med. 2007;14(2):97-107.
5. Fergusson DM, Woodward LJ. Mental health, educational, and social role outcomes of adolescents with depression. Arch Gen Psychiatry. 2002;59(3):225-231.
6. Gould MS, King R, Greenwald S, Fisher P, Schwab-Stone M, Kramer R, et al. Psychopathology associated with suicidal ideation and attempts among children and adolescents. J Am Acad Child Adolesc Psychiatry. 1998;37(9):915-923.
7. Fergusson DM, Boden JM, Horwood LJ. Recurrence of major depression in adolescence and early adulthood, and later mental health, educational and economic outcomes. Br J Psychiatry. 2007;191:335-342.
8. Lewinsohn PM, Rohde P, Klein DN, Seeley JR. Natural course of adolescent major depressive disorder: I. Continuity into young adulthood. J Am Acad Child Adolesc Psychiatry. 1999;38(1):56-63.
9. Curry J, Silva S, Rohde P, Ginsburg G, Kratochvil C, Simons A, et al. Recovery and recurrence following treatment for adolescent major depression. Arch Gen Psychiatry. 2011;68(3):263-269.
10. Gulliver A, Griffiths KM, Christensen H. Perceived barriers and facilitators to mental health help-seeking in young people: a systematic review. BMC Psychiatry. 2010;10:113.
11. American College Health Association-National College Health Assessment Spring 2008 Reference Group Data Report (abridged): the American College Health Association. J Am Coll Health. 2009;57(5):477-488.
12. Franzen PL, Buysse DJ. Sleep disturbances and depression: risk relationships for subsequent depression and therapeutic implications. Dialogues Clin Neurosci. 2008;10(4):473-481.
13. Fernandez-Mendoza J, Vgontzas AN. Insomnia and its impact on physical and mental health. Curr Psychiatry Rep. 2013;15(12):418.
14. Luo C, Zhang J, Pan J. One-year course and effects of insomnia in rural Chinese adolescents. Sleep. 2013;36(3):377-384.
15. Baglioni C, Battagliese G, Feige B, Spiegelhalder K, Nissen C, Voderholzer U, et al. Insomnia as a predictor of depression: a meta-analytic evaluation of longitudinal epidemiological studies. J Affect Disord. 2011;135(1-3):10-19.
16. Manglick M, Rajaratnam SM, Taffe J, Tonge B, Melvin G. Persistent sleep disturbance is associated with treatment response in adolescents with depression. Aust N Z

J Psychiatry. 2013;47(6):556-563.
17. Li S, Chan J, Lam J, Yu M, Wing Y. Can nocturnal sleep disturbances predict non-remission and relapse in patients with major depressive disorder? VA 5-year naturalistic longitudinal study. Sleep Med. 2013;14:e48.
18. Li SX, Lam SP, Chan JW, Yu MW, Wing YK. Residual sleep disturbances in patients remitted from major depressive disorder: a 4-year naturalistic follow-up study. Sleep. 2012;35(8):1153-1161.
19. Li SX, Lam SP, Yu MW, Zhang J, Wing YK. Nocturnal sleep disturbances as a predictor of suicide attempts among psychiatric outpatients: a clinical, epidemiologic, prospective study. J Clin Psychiatry. 2010;71(11):1440-1446.
20. Pigeon WR, Pinquart M, Conner K. Meta-analysis of sleep disturbance and suicidal thoughts and behaviors. The Journal of clinical psychiatry. 2012;73(9):e1160-1167.
21. Bonnet MH, Arand DL. Hyperarousal and insomnia: state of the science. Sleep Med Rev. 2010;14(1):9-15.
22. Riemann D, Spiegelhalder K, Feige B, Voderholzer U, Berger M, Perlis M, et al. The hyperarousal model of insomnia: a review of the concept and its evidence. Sleep Med Rev. 2010;14(1):19-31.
23. Rodenbeck A, Huether G, Ruther E, Hajak G. Interactions between evening and nocturnal cortisol secretion and sleep parameters in patients with severe chronic primary insomnia. Neurosci Lett. 2002;324(2):159-1563.
24. Vgontzas AN, Bixler EO, Lin HM, Prolo P, Mastorakos G, Vela-Bueno A, et al. Chronic insomnia is associated with nyctohemeral activation of the hypothalamic-pituitary-adrenal axis: clinical implications. J Clin Endocrinol Metab. 2001;86(8):3787-3794.
25. Young E, Korszun A. Sex, trauma, stress hormones and depression. Mol Psychiatry. 2010;15(1):23-28.
26. Staner L. Comorbidity of insomnia and depression. Sleep Med Rev. 2010;14(1):35-46.
27. Staner L, Duval F, Haba J, Mokrani MC, Macher JP. Disturbances in hypothalamo pituitary adrenal and thyroid axis identify different sleep EEG patterns in major depressed patients. J Psychiatr Res. 2003;37(1):1-8.
28. Vgontzas AN, Tsigos C, Bixler EO, Stratakis CA, Zachman K, Kales A, et al. Chronic insomnia and activity of the stress system: a preliminary study. J Psychosom Res. 1998;45(1):21-31.
29. Blake MJ, Trinder JA, Allen NB. Mechanisms underlying the association between insomnia, anxiety, and depression in adolescence: Implications for behavioral sleep interventions. Clin Psychol Rev. 2018;63:25-40.
30. Buysse DJ, Angst J, Gamma A, Ajdacic V, Eich D, Rossler W. Prevalence, course, and comorbidity of insomnia and depression in young adults. Sleep. 2008;31(4):473-480.
31. Johnson EO, Roth T, Breslau N. The association of insomnia with anxiety disorders and depression: exploration of the direction of risk. J Psychiatr Res. 2006;40(8):700-708.
32. Roane BM, Taylor DJ. Adolescent insomnia as a risk factor for early adult depression and substance abuse. Sleep. 2008;31(10):1351-1356.
33. Roberts RE, Shema SJ, Kaplan GA, Strawbridge WJ. Sleep complaints and depression in an aging cohort: A prospective perspective. Am J Psychiatry. 2000;157(1):81-88.

34. Morin CM, Bootzin RR, Buysse DJ, Edinger JD, Espie CA, Lichstein KL. Psychological and behavioral treatment of insomnia:update of the recent evidence (1998-2004). Sleep. 2006;29(11):1398-414.
35. Wu JQ, Appleman ER, Salazar RD, Ong JC. Cognitive Behavioral Therapy for Insomnia Comorbid With Psychiatric and Medical Conditions: A Meta-analysis. JAMA Intern Med. 2015;175(9):1461-1472.
36. Smith MT, Perlis ML, Park A, Smith MS, Pennington J, Giles DE, et al. Comparative meta-analysis of pharmacotherapy and behavior therapy for persistent insomnia. Am J Psychiatry. 2002;159(1):5-11.
37. Riemann D, Nissen C, Palagini L, Otte A, Perlis ML, Spiegelhalder K. The neurobiology, investigation, and treatment of chronic insomnia. Lancet Neurol. 2015;14(5):547-558.
38. Friedrich A, Schlarb AA. Let's talk about sleep: a systematic review of psychological interventions to improve sleep in college students. J Sleep Res. 2018;27(1):4-22.
39. Yi MJ, Kim TW, Jeong JH, Joo SH, Hong SC, Suh SY. Case Studies of Chronic Insomnia Patients Participating in Group Cognitive Behavioral Therapy for Insomnia. Sleep Med Res. 2012;3(2):45-49.
40. Everitt H, McDermott L, Leydon G, Yules H, Baldwin D, Little P. GPs' management strategies for patients with insomnia: a survey and qualitative interview study. Br J Gen Pract. 2014;64(619):e112-119.
41. van der Zweerde T, Lancee J, Slottje P, Bosmans J, Van Someren E, Reynolds C, 3rd, et al. Cost-effectiveness of i-Sleep, a guided online CBT intervention, for patients with insomnia in general practice: protocol of a pragmatic randomized controlled trial. BMC Psychiatry. 2016;16:85.
42. Zachariae R, Lyby MS, Ritterband LM, O'Toole MS. Efficacy of internet-delivered cognitive-behavioral therapy for insomnia - A systematic review and meta-analysis of randomized controlled trials. Sleep Med Rev. 2016;30:1-10.
43. Koffel EA, Koffel JB, Gehrman PR. A meta-analysis of group cognitive behavioral therapy for insomnia. Sleep Med Rev. 2015;19:6-16.
44. de Bruin EJ, Bögels SM, Oort FJ, Meijer AM. Efficacy of Cognitive Behavioral Therapy for Insomnia in Adolescents: A Randomized Controlled Trial with Internet Therapy, Group Therapy and A Waiting List Condition. Sleep. 2015;38(12):1913-1926.
45. de Bruin EJ, Oort FJ, Bogels SM, Meijer AM. Efficacy of internet and group-administered cognitive behavioral therapy for insomnia in adolescents: a pilot study. Behav Sleep Med. 2014;12(3):235-254.
46. De Bruin EJ, van Steensel FJ, Meijer AM. Cost-Effectiveness of Group and Internet Cognitive Behavioral Therapy for Insomnia in Adolescents: Results from a Randomized Controlled Trial. Sleep. 2016;39(8):1571-1581.
47. Richardson T, Stallard P, Velleman S. Computerised cognitive behavioral therapy for the prevention and treatment of depression and anxiety in children and adolescents: a systematic review. Clin Child Fam Psychol Rev. 2010;13(3):275-290.
48. King R, Bambling M, Lloyd C, Gomurra R, Smith S, Reid W, et al. Online counselling: The motives and experiences of young people who choose the Internet instead of face to face or telephone counselling. Couns Psychother Res. 2006;6(3):169-174.
49. Burns JM, Davenport TA, Durkin LA, Luscombe GM, Hickie IB. The internet as a

setting for mental health service utilisation by young people. Med J Aust. 2010;192(11 Suppl):S22-26.
50. Christensen H, Batterham PJ, Gosling JA, Ritterband LM, Griffiths KM, Thorndike FP, et al. Effectiveness of an online insomnia program (SHUTi) for prevention of depressive episodes (the GoodNight Study): a randomised controlled trial. Lancet Psychiatry. 2016;3(4):333-341.
51. Cheng P, Kalmbach DA, Tallent G, Joseph CL, Espie CA, Drake CL. Depression prevention via digital cognitive behavioral therapy for insomnia: a randomized controlled trial. Sleep. 2019;42(10).
52. Espie CA, Emsley R, Kyle SD, Gordon C, Drake CL, Siriwardena AN, et al. Effect of Digital Cognitive Behavioral Therapy for Insomnia on Health, Psychological Well-being, and Sleep-Related Quality of Life: A Randomized Clinical Trial. JAMA psychiatry. 2018.
53. Freeman D, Sheaves B, Goodwin GM, Yu LM, Nickless A, Harrison PJ, et al. The effects of improving sleep on mental health (OASIS): a randomised controlled trial with mediation analysis. Lancet Psychiatry. 2017;4(10):749-758.
54. Morris J, Firkins A, Millings A, Mohr C, Redford P, Rowe A. Internet-delivered cognitive behavior therapy for anxiety and insomnia in a higher education context. Anxiety Stress Coping. 2016;29(4):415-431.
55. Kroenke K, Spitzer RL, Williams JB. The PHQ-9: validity of a brief depression severity measure. J Gen Intern Med. 2001;16(9):606-613.
56. Bastien CH, Vallières A, Morin CM. Validation of the Insomnia Severity Index as an outcome measure for insomnia research. Sleep Med. 2001;2(4):297-307.
57. World Health Organization. The ICD-10 classification of mental and behavioral disorders: clinical descriptions and diagnostic guidelines: Geneva: World Health Organization; 1992.
58. Sheehan DV, Lecrubier Y, Sheehan KH, Amorim P, Janavs J, Weiller E, et al. The Mini-International Neuropsychiatric Interview (M.I.N.I.): the development and validation of a structured diagnostic psychiatric interview for DSM-IV and ICD-10. J Clin Psychiatry. 1998;59 Suppl 20:22-33;quiz 4-57.
59. Wing YK, Li AM, Lam JS, Li SX, Kwok AP. Brief integrated sleep-focused treatment for persistent sleep disturbance in residual depression: an assessor-blind, parallel group, randomised controlled study. Hong Kong Med J. 2018;24 Suppl 2(1):8-11.
60. Spitzer RL, Kroenke K, Williams JB, Löwe B. A brief measure for assessing generalized anxiety disorder: the GAD-7. Arch Intern Med. 2006;166(10):1092-1097.
61. Beck AT, Steer RA, Ranieri WF. Scale for Suicide Ideation: psychometric properties of a self-report version. J Clin Psychol. 1988;44(4):499-505.
62. Smets E, Garssen B, Bonke Bd, De Haes J. The Multidimensional Fatigue Inventory (MFI) psychometric qualities of an instrument to assess fatigue. J Psychosom. Res. 1995;39(3):315-325.
63. Adan A, Almirall H. Horne & Östberg morningness-eveningness questionnaire: A reduced scale. Pers Individ Differ 1991;12(3):241-253.
64. Morin CM, Vallières A, Ivers H. Dysfunctional beliefs and attitudes about sleep (DBAS): validation of a brief version (DBAS-16). Sleep. 2007;30(11):1547-1554.
65. Kyle SD, Morgan K, Spiegelhalder K, Espie CA. No pain, no gain: an exploratory within-subjects mixed-methods evaluation of the patient experience of sleep restriction therapy (SRT) for insomnia. Sleep Med. 2011;12(8):735-747.

Appendix: Amendment history

Amendment No.	Protocol Version No.	Date of issue	Details of Changes made
1	2.0	24/10/2019	Broadened the age range of participants from 18-25 years to 15-25 years to examine the effectiveness of e-CBT-I in a wider population. Wording clarification of the study population being youth population instead of college students.
			Wording clarification of the assessments time points.
			Addition of saliva collection, which had to be discontinued later due to COVID-19 pandemic.
			Addition to inclusion and exclusion criteria for clarification
			Wording clarification of diagnostic criteria of insomnia disorder and outcome measures.
			Addition of a description of the application control management system for e-CBT-I.
			Correction of typographic errors.
2	3.0	28/08/2020	Addition of recruitment methods to identify potential participants.
3	4.0	07/01/2022	Adding a focus group to investigate the user experience and satisfaction towards e-CBT-I intervention.
			Reduced the total sample size from 940 to 708 for a lower attrition rate than expected.
4	5.0	08/12/2022	Addition to assess interrater reliability.
			Correction of typographic errors.

All amendments to the original protocol were approved by the Joint Chinese University of Hong Kong-New Territories East Cluster Clinical Research Ethics Committee (CREC Ref. No.: 2019.044).
