# Supplementary material for: Effectiveness of app-based cognitive behavioral therapy for insomnia on preventing major depressive disorder in youth with insomnia and subclinical depression: A randomized clinical trial
Source: PLoS Med. 2025 Jan 21;22(1):e1004510. doi: 10.1371/journal.pmed.1004510 (PMC11750088; doi:10.1371/journal.pmed.1004510)
Supplement: S1 Appendix — Table A. Description and completion rates of app-based CBT-I and app-based health education (HE) sessions during the intervention period. Table B. Hazard ratio (HR) of incident major depression in app-based CBT-I group as compared to app-based HE. Table C. HR of incident major depression in app-based CBT-I group as compared to app-based HE among participants with persistent insomnia. Table D. Secondary outcomes with imputed missing data due to participants reaching study endpoint before final follow-up. Table E. Mediation analysis results. Table F. Other prespecified outcomes. Table G. Other prespecified outcomes with imputed missing data due to participants reaching study endpoint before final follow-up. Fig A. Sleep-promoting medication use by intervention group. Fig B. Risk of developing major depressive disorder by subgroups. Fig C. Risk of developing major depressive disorder by subgroups among participants with persistent insomnia. Fig D. Remission rates of insomnia disorder by intervention group with imputed missing data due to participants reaching study endpoint before final follow-up. Fig E. Comparison of secondary outcomes at each assessment. Fig F. Comparison of other prespecified outcomes at each assessment. (DOCX) [file pmed.1004510.s003.docx]

**Supplementary Appendix**

**Effectiveness of App-based Cognitive Behavioral Therapy for Insomnia on Preventing Major Depressive Disorder in Youth with Insomnia and Subclinical Depression: A Randomized Controlled Trial**

| **Table A** | Description and completion rates of app-based CBT-I and app-based HE sessions during the intervention period |
| --- | --- |
| **Table B** | Hazard ratio of incident major depression in app-based CBT-I group as compared to app-based HE |
| **Table C** | Hazard ratio of incident major depression in app-based CBT-I group as compared to app-based HE among participants with persistent insomnia |
| **Table D** | Secondary outcomes with imputed missing data due to participants reaching study endpoint before final follow-up |
| **Table E** | Mediation analysis results |
| **Table F** | Other prespecified outcomes |
| **Table G** | Other prespecified outcomes with imputed missing data due to participants reaching study endpoint before final follow-up |
| **Fig A** | Sleep-promoting medication use by intervention group |
| **Fig B** | Risk of developing major depressive disorder by subgroups |
| **Fig C** | Risk of developing major depressive disorder by subgroups among participants with persistent insomnia |
| **Fig D** | Remission rates of insomnia disorder by intervention group with imputed missing data due to participants reaching study endpoint before final follow-up |
| **Fig E** | Comparison of secondary outcomes at each assessment |
| **Fig F** | Comparison of other prespecified outcomes at each assessment |

**Table A. Description and completion rates of app-based CBT-I and app-based HE sessions during the intervention period**

| Session | App-based CBT-I | No. of participants in app-based CBT-I group who completed the session (n = 354) | App-based HE | No. of participants in app-based HE group who completed the session (n = 354) |
| --- | --- | --- | --- | --- |
| Session 1 | Overview of the program and psychoeducation about sleep and insomnia | 333 (94%) | Overview of the program and introduction of general knowledge about sleep (e.g., stages of sleep, homeostatic sleep drive and circadian rhythm) and sleep hygiene education (i.e., maintaining a comfortable sleep environment, setting a regular sleep schedule, limiting food and caffeine intake, and following a bedtime routine) | 332 (94%) |
| Session 2 | Introduction of sleep restriction technique; review of sleep diary, setting sleep window, and introduction of strategies to implement sleep restriction, stress management and breathing exercises for relaxation | 318 (90%) | Introduction of general knowledge about elements of nutrition (i.e., carbohydrates, proteins, fats, and vitamins) | 326 (92%) |
| Session 3 | Review of sleep diary and adjusting sleep window time; review of sleep restriction with an emphasis on the importance of the regularity of sleep-wake pattern; introduction of stimulus control strategies; overview of good and bad sleep hygiene practice; and introduction of progressive muscle relaxation | 309 (87%) | Introduction of functions of human organs (i.e., heart, pancreas, skin, liver, lung, and kidney) | 315 (89%) |
| Session 4 | Review of sleep diary and adjusting sleep window time; review of sleep restriction and stimulus control strategies; and introduction of cognitive restructuring and guided imagery | 303 (86%) | Introduction of environmental pollutions (i.e., smog, noise pollution, water pollution, light pollution, and radiation) and their associations with health and sleep | 308 (87%) |

**Table A. Description and completion rates of app-based CBT-I and app-based HE sessions during the intervention period (continued)**

| Session 5 | Review of sleep diary and adjusting sleep window time; and introduction of constructive worry technique and meditation | 301 (85%) | Introduction of general knowledge about brain health | 304 (86%) |
| --- | --- | --- | --- | --- |
| Session 6 | Review of the program and relapse prevention | 296 (84%) | Identification and treatment of common diseases (i.e., allergies, heart disease, colorectal cancer, gastritis, hypertension, and chronic obstructive pulmonary disease) | 301 (85%) |

Abbreviations: CBT-I, cognitive behavioral therapy for insomnia; HE, health education.

Note: The number of sessions completed by participants was recorded by the application control management system.

**Table B. Hazard ratio of incident major depression in app-based CBT-I group as compared to app-based HE**

| Variables | Model 1 |  |  | Model 2 |  | |
| --- | --- | --- | --- | --- | --- | --- |
|  | HR | 95%CI |  | HR | 95%CI | |
| App-based CBT-I intervention (ref. app-based HE) | 0.58** | 0.38, 0.87 |  | 0.60* | 0.40, 0.90 | |
| Sex (ref. female) | 0.44*** | 0.28, 0.70 |  | 0.46** | 0.28, 0.73 | |
| Insomnia severity (per point) | 1.10** | 1.03, 1.18 |  | 1.12** | 1.04, 1.20 | |
| History of MDD (ref. none) | NA | NA |  | 2.08** | 1.37, 3.17 | |
| Educational level (ref. undergraduate student and above) | NA | NA |  | 0.93 | 0.42, 2.06 | |
| Family income (ref. CNY < 8000) | NA | NA |  | 1.45 | 0.92, 2.22 | |
| Diagnosed medical illnesses^a^ (ref. none) | NA | NA |  | 1.12 | 0.70, 1.70 | |
| Use of sleep-promoting medications at baseline^b^ (ref. none) | NA | NA |  | 0.63 | 0.38, 1.20 | |
| Abbreviations: CBT-I, cognitive behavioral therapy for insomnia; HE, health education; HR, hazard ratio.  ^a^ Medical illnesses included eye disease, arthritis, heart disease, diabetes, renal disease, gastro-oesophageal reflux disease, hypertension, epilepsy, chronic lung disease and chronic pain.  ^b^ Sleep-promoting medications included benzodiazepines, non-benzodiazepines, trazodone (25-50mg), traditional Chinese medicine, sedating antihistamines and melatonin.  *** *p* < 0.001; ** *p* < 0.01; * *p* < 0.05. | | | | | |  |

**Table C. Hazard ratio of incident major depression in app-based CBT-I group as compared to app-based HE among participants with persistent insomnia**

| Variables | Model 1 |  |  | Model 2 |  | |
| --- | --- | --- | --- | --- | --- | --- |
|  | HR | 95%CI |  | HR | 95%CI | |
| App-based CBT-I intervention (ref. app-based HE) | 0.56* | 0.36, 0.88 |  | 0.68* | 0.37, 0.90 | |
| Sex (ref. female) | 0.45** | 0.28, 0.75 |  | 0.47** | 0.28, 0.78 | |
| Insomnia severity (per point) | 1.09* | 1.01, 1.18 |  | 1.10* | 1.02, 1.19 | |
| History of MDD (ref. none) | NA | NA |  | 2.03** | 1.28, 3.22 | |
| Educational level (ref. undergraduate student and above) | NA | NA |  | 0.97 | 0.42, 2.32 | |
| Family income (ref. CNY < 8000) | NA | NA |  | 1.28 | 0.80, 2.04 | |
| Diagnosed medical illnesses^a^ (ref. none) | NA | NA |  | 1.21 | 0.72, 2.02 | |
| Use of sleep-promoting medications at baseline^b^ (ref. none) | NA | NA |  | 0.87 | 0.49, 1.57 | |
| Abbreviations: CBT-I, cognitive behavioral therapy for insomnia; HE, health education; HR, hazard ratio.  ^a^ Medical illnesses included eye disease, arthritis, heart disease, diabetes, renal disease, gastro-oesophageal reflux disease, hypertension, epilepsy, chronic lung disease and chronic pain.  ^b^ Sleep-promoting medications included benzodiazepines, non-benzodiazepines, trazodone (25-50mg), traditional Chinese medicine, sedating antihistamines and melatonin.  ** *p* < 0.01; * *p* < 0.05. | | | | | |  |

**Table D. Secondary outcomes with imputed missing data** **due to participants reaching study endpoint before final follow-up**

|  | App-based CBT-I, mean (SE)^a^ | App-based HE, mean (SE) | | Adjusted difference  (95% CI) | | *p* value | Cohen’s *d*^b^ |
| --- | --- | --- | --- | --- | --- | --- | --- |
|  |  |  |  |  |  |  |  |
| *PHQ-9* | |  | |  | |  |  |
| Baseline | 12.1 (0.2) | 12.1 (0.2) | | .. | | .. | .. |
| Post-session 2 | 7.6 (0.2) | 7.7 (0.2) | | -0.1 (-0.7, 0.5) | | 0.73 | 0.05 |
| Post-session 4 | 6.1 (0.2) | 7.0 (0.2) | | -0.9 (-1.5, -0.3) | | 0.003 | 0.49 |
| Post-intervention | 5.1 (0.2) | 6.2 (0.2) | | -1.0 (-1.6, -0.5) | | < 0.001 | 0.54 |
| 6-month | 5.5 (0.2) | 6.4 (0.2) | | -0.9 (-1.5, -0.3) | | 0.004 | 0.46 |
| 12-month | 5.6 (0.2) | 6.5 (0.2) | | -0.8 (-1.4, -0.2) | | 0.01 | 0.40 |
| *PHQ-8^c^* |  |  | |  | |  |  |
| Baseline | 9.6 (0.2) | 9.6 (0.2) | | .. | | .. | .. |
| Post-session 2 | 6.2 (0.2) | 6.2 (0.2) | | -0.1 (-0.6, 0.5) | | 0.85 | 0.03 |
| Post-session 4 | 4.9 (0.2) | 5.6 (0.2) | | -0.7 (-1.2, -0.1) | | 0.02 | 0.38 |
| Post-intervention | 4.2 (0.2) | 5.0 (0.2) | | -0.9 (-1.4, -0.4) | | 0.001 | 0.51 |
| 6-month | 4.5 (0.2) | 5.3 (0.2) | | -0.8 (-1.3, -0.2) | | 0.006 | 0.45 |
| 12-month | 4.7 (0.2) | 5.3 (0.2) | | -0.7 (-1.2, -0.1) | | 0.02 | 0.38 |
| *ISI* | |  |  | |  |  |  |
| Baseline | 18.3 (0.2) | 18.3 (0.2) | | .. | | .. | .. |
| Post-session 2 | 11.5 (0.2) | 12.1 (0.2) | | -0.5 (-1.2, 0.2) | | 0.13 | 0.22 |
| Post-session 4 | 9.0 (0.2) | 10.7 (0.2) | | -1.7 (-2.4, -0.9) | | < 0.001 | 0.67 |
| Post-intervention | 7.3 (0.2) | 9.3 (0.2) | | -2.0 (-2.7, -1.3) | | < 0.001 | 0.79 |
| 6-month | 7.3 (0.2) | 8.6 (0.2) | | -1.3 (-2.0, -0.6) | | < 0.001 | 0.52 |
| 12-month | 7.5 (0.2) | 8.7 (0.2) | | -1.2 (-1.9, -0.4) | | 0.002 | 0.46 |
| Abbreviations: CBT-I, based cognitive behavioral therapy for insomnia; HE, based health education; ISI, Insomnia Severity Index; PHQ, Patient Health Questionnaire.  ^a^ All values are adjusted for sex and insomnia severity at baseline.  ^b^ Cohen’s *d* is defined as the adjusted intervention effect divided by the square root of estimated sample variance derived from the linear mixed-effects model.  ^c^ Excluding sleep item. | | | | | | |  |

**Table E. Mediation analysis results**

|  |  | Total effect |  |  | Direct effect | |  | Indirect effect | |  |
| --- | --- | --- | --- | --- | --- | --- | --- | --- | --- | --- |
|  | Mediation tested | Effect size (SE) | *p* |  | Effect size (SE) | *p* |  | Effect size (SE) | *p* | Mediation |
| *PHQ-8^a^* |  |  |  |  |  |  |  |  |  |  |
| Post-intervention | ISI at post-session 4 | -0.88 (0.26) | 0.001 |  | -0.22 (0.24) | 0.35 |  | -0.65 (0.14) | < 0.001 | 74% |
| 6-momth | ISI at post-intervention | -0.64 (0.29) | 0.03 |  | -0.14 (0.28) | 0.60 |  | -0.50 (0.11) | < 0.001 | 78% |
| *ISI* |  |  |  |  |  |  |  |  |  |  |
| Post-intervention | PHQ-8 at post-session 4 | -2.03 (0.36) | < 0.001 |  | -1.64 (0.33) | < 0.001 |  | -0.39 (0.17) | 0.02 | 19% |
| 6-momth | PHQ-8 at post-intervention | -1.22 (0.38) | 0.001 |  | -0.70 (0.36) | 0.04 |  | -0.52 (0.16) | 0.001 | 43% |
| Abbreviations: ISI, Insomnia Severity Index; PHQ, Patient Health Questionnaire.  Models were adjusted for sex and baseline insomnia severity.  ^a^ Excluding sleep item. | | | | | | | | |  |  |

**Table F. Other prespecified outcomes**

|  | App-based CBT-I, mean (SE)^a^ | App-based HE, mean (SE) | Adjusted difference  (95% CI) | *p* value | Cohen’s *d*^b^ |
| --- | --- | --- | --- | --- | --- |
|  |  |  |  |  |  |
| *Sleep onset latency (min)* | |  |  |  |  |
| Baseline | 53.2 (1.8) | 52.6 (1.8) | .. | .. | .. |
| Post-session 2 | 41.1 (1.8) | 44.6 (1.9) | -4.1 (-9.3, 1.1) | 0.12 | 0.24 |
| Post-session 4 | 33.6 (1.9) | 40.5 (1.9) | -7.5 (-12.9, -2.0) | 0.007 | 0.44 |
| Post-intervention | 29.2 (1.8) | 39.8 (1.8) | -11.2 (-16.3, -6.1) | < 0.001 | 0.67 |
| 6-month | 29.5 (1.9) | 36.9 (1.9) | -8.0 (-13.4, -2.5) | 0.004 | 0.48 |
| 12-month | 31.2 (2.0) | 34.1 (2.1) | -3.5 (-9.2, 2.2) | 0.23 | 0.21 |
| *Wake after sleep onset (min)* | |  |  |  |  |
| Baseline | 8.9 (0.5) | 8.8 (0.6) | .. | .. | .. |
| Post-session 2 | 6.7 (0.6) | 7.9 (0.6) | -1.4 (-3.0, 0.3) | 0.10 | 0.26 |
| Post-session 4 | 5.7 (0.6) | 8.1 (0.6) | -2.4 (-4.1, -0.8) | 0.005 | 0.47 |
| Post-intervention | 5.3 (0.6) | 6.0 (0.6) | -0.8 (-2.4, 0.8) | 0.34 | 0.15 |
| 6-month | 4.6 (0.6) | 5.0 (0.6) | -0.6 (-2.3, 1.1) | 0.49 | 0.12 |
| 12-month | 4.9 (0.6) | 4.9 (0.6) | -0.1 (-1.9, 1.6) | 0.87 | 0.03 |
| *Total sleep time (min)* |  |  |  |  |  |
| Baseline | 401.1 (3.0) | 394.4 (3.0) | .. | .. | .. |
| Post-session 2 | 408.7 (3.1) | 409.6 (3.2) | -7.6 (-16.9, 1.6) | 0.11 | 0.24 |
| Post-session 4 | 419.5 (3.3) | 414.9 (3.3) | -2.1 (-11.8, -7.5) | 0.66 | 0.07 |
| Post-intervention | 429.6 (3.1) | 420.8 (3.1) | 2.1 (-7.0, 11.2) | 0.65 | 0.07 |
| 6-month | 427.3 (3.3) | 423.4 (3.3) | -2.9 (-12.5, 6.8) | 0.56 | 0.09 |
| 12-month | 436.7 (3.4) | 430.5 (3.5) | -0.6 (-10.8, 9.5) | 0.90 | 0.02 |
| *Time in bed (min)* |  |  |  |  |  |
| Baseline | 500.8 (3.2) | 493.6 (3.2) | .. | .. | .. |
| Post-session 2 | 483.7 (3.4) | 491.6 (3.4) | -15.2 (-25.0, -5.4) | 0.002 | 0.47 |
| Post-session 4 | 480.5 (3.6) | 490.1 (3.6) | -16.9 (-27.1, -6.7) | 0.001 | 0.52 |
| Post-intervention | 485.0 (3.4) | 490.9 (3.3) | -13.2 (-22.8, -3.6) | 0.007 | 0.41 |
| 6-month | 482.6 (3.6) | 488.9 (3.6) | -13.5 (-23.7, -3.3) | 0.01 | 0.42 |
| 12-month | 493.4 (3.7) | 495.6 (3.8) | -9.5 (-20.2, 1.2) | 0.08 | 0.29 |
| *Sleep efficiency, %* |  |  |  |  |  |
| Baseline | 80.5 (0.4) | 80.3 (0.4) | .. | .. | .. |
| Post-session 2 | 84.9 (0.5) | 83.5 (0.5) | 1.2 (-0.1, 2.5) | 0.06 | 0.30 |
| Post-session 4 | 87.7 (0.5) | 84.9 (0.5) | 2.7 (1.3, 4.0) | < 0.001 | 0.64 |
| Post-intervention | 89.0 (0.5) | 86.0 (0.5) | 2.8 (1.5, 4.1) | < 0.001 | 0.68 |
| 6-month | 88.7 (0.5) | 86.9 (0.5) | 1.6 (0.3, 3.0) | 0.02 | 0.39 |
| 12-month | 88.8 (0.5) | 87.0 (0.5) | 1.6 (0.2, 3.0) | 0.03 | 0.39 |
| *GAD-7* |  |  |  |  |  |
| Baseline | 7.7 (0.2) | 7.9 (0.2) | .. | .. | .. |
| Post-session 2 | 5.8 (0.2) | 6.2 (0.2) | -0.3 (-0.9, 0.3) | 0.40 | 0.14 |
| Post-session 4 | 4.6 (0.2) | 5.6 (0.2) | -0.8 (-1.4, -0.2) | 0.01 | 0.42 |
| Post-intervention | 4.0 (0.2) | 4.9 (0.2) | -0.7 (-1.3, -0.1) | 0.02 | 0.38 |
| 6-month | 4.3 (0.2) | 5.0 (0.2) | -0.5 (-1.1, 0.1) | 0.10 | 0.27 |
| 12-month | 4.4 (0.2) | 4.8 (0.2) | -0.3 (-0.9, 0.4) | 0.41 | 0.14 |
| *SSI-C^c^* |  |  |  |  |  |
| Baseline | 0.17 (0.02) | 0.15 (0.02) | .. | .. |  |
| Post-intervention | 0.12 (0.02) | 0.13 (0.02) | -0.03 (-0.08, 0.02) | 0.24 | 0.17 |
| 6-month | 0.12 (0.02) | 0.10 (0.02) | 0.00 (-0.06, 0.05) | 0.89 | 0.02 |
| 12-month | 0.12 (0.02) | 0.11 (0.02) | -0.01 (-0.06, 0.05) | 0.85 | 0.03 |
| *MFI* |  |  |  |  |  |
| Baseline | 65.9 (0.8) | 64.7 (0.8) | .. | .. | .. |
| Post-intervention | 53.7 (0.8) | 57.3 (0.8) | -4.8 (-7.0, -2.6) | < 0.001 | 0.70 |
| 6-month | 52.2 (0.9) | 55.4 (0.9) | -4.4 (-6.7, -2.1) | < 0.001 | 0.64 |
| 12-month | 53.5 (0.9) | 55.8 (0.9) | -3.5 (-5.9, -1.0) | 0.005 | 0.50 |

**Table F. Other prespecified outcomes (continued)**

| *rMEQ* |  |  |  |  |  |
| --- | --- | --- | --- | --- | --- |
| Baseline | 12.3 (0.2) | 12.4 (0.2) | .. | .. | .. |
| Post-intervention | 13.7 (0.2) | 13.1 (0.2) | 0.7 (0.2 to 1.1) | 0.002 | 0.59 |
| 6-month | 13.6 (0.2) | 13.1 (0.2) | 0.6 (0.1 to 1.0) | 0.01 | 0.51 |
| 12-month | 13.6 (0.2) | 12.9 (0.2) | 0.8 (0.3 to 1.2) | 0.001 | 0.68 |
| *DBAS-16* |  |  |  |  |  |
| Baseline | 7.1 (0.1) | 7.1 (0.1) | .. | .. | .. |
| Post-intervention | 5.4 (0.1) | 6.6 (0.1) | -1.2 (-1.4 to -1.0) | < 0.001 | 1.79 |
| 6-month | 5.5 (0.1) | 6.3 (0.1) | -0.8 (-1.0 to -0.6) | < 0.001 | 1.20 |
| 12-month | 5.6 (0.1) | 6.3 (0.1) | -0.7 (-0.9 to -0.5) | < 0.001 | 1.05 |

Abbreviations: CBT-I, cognitive behavioral therapy for insomnia; DBAS-16, brief version of Dysfunctional Beliefs and Attitudes about Sleep; GAD-7, Generalized Anxiety Disorder 7-item; HE, health education; MFI, Multidimensional Fatigue Inventory; rMEQ, reduced Horne and Östberg Morningness−Eveningness Questionnaire; SSI-C, Scale for Suicide Ideation-Current.

^a^ All values are adjusted for sex and insomnia severity at baseline.

^b^ Cohen’s *d* is defined as the adjusted intervention effect divided by the square root of estimated sample variance derived from the linear mixed-effects model.

^c^ Data are log-transformed prior to analysis to achieve near-normal distribution.

**Table G. Other prespecified outcomes with imputed missing data due to participants reaching study endpoint before final follow-up**

|  | App-based CBT-I, mean (SE)^a^ | App-based HE, mean (SE) | Adjusted difference  (95% CI) | *p* value | Cohen’s *d*^b^ |
| --- | --- | --- | --- | --- | --- |
|  |  |  |  |  |  |
| *Sleep onset latency (min)* | |  |  |  |  |
| Baseline | 53.2 (1.8) | 52.6 (1.8) | .. | .. | .. |
| Post-session 2 | 41.0 (1.8) | 44.6 (1.9) | -4.1 (-9.3, 1.1) | 0.12 | 0.24 |
| Post-session 4 | 33.6 (1.9) | 40.6 (1.9) | -7.5 (-12.9, -2.0) | 0.007 | 0.44 |
| Post-intervention | 29.2 (1.8) | 39.8 (1.8) | -11.2 (-16.3, -6.1) | < 0.001 | 0.66 |
| 6-month | 29.4 (1.9) | 37.5 (1.9) | -8.6 (-14.0, -3.2) | 0.002 | 0.51 |
| 12-month | 30.9 (2.0) | 35.7 (2.0) | -5.4 (-10.9, 0.2) | 0.06 | 0.31 |
| *Wake after sleep onset (min)* | |  |  |  |  |
| Baseline | 8.9 (0.5) | 8.8 (0.6) | .. | .. | .. |
| Post-session 2 | 6.7 (0.6) | 7.9 (0.6) | -1.3 (-2.9, 0.3) | 0.11 | 0.25 |
| Post-session 4 | 5.8 (0.6) | 8.1 (0.6) | -2.4 (-4.1, -0.7) | 0.005 | 0.47 |
| Post-intervention | 5.3 (0.6) | 6.0 (0.6) | -0.8 (-2.4, 0.8) | 0.34 | 0.15 |
| 6-month | 4.6 (0.6) | 5.1 (0.6) | -0.6 (-2.3, 1.0) | 0.46 | 0.12 |
| 12-month | 4.9 (0.6) | 5.0 (0.6) | -0.2 (-2.0, 1.5) | 0.78 | 0.05 |
| *Total sleep time (min)* |  |  |  |  |  |
| Baseline | 401.1 (3.0) | 394.4 (3.0) | .. | .. | .. |
| Post-session 2 | 408.7 (3.1) | 409.7 (3.2) | -7.7 (-16.9, 1.5) | 0.10 | 0.25 |
| Post-session 4 | 419.6 (3.3) | 414.8 (3.3) | -2.0 (-11.6, 7.6) | 0.68 | 0.07 |
| Post-intervention | 429.6 (3.1) | 420.8 (3.1) | 2.1 (-7.0, 11.1) | 0.65 | 0.07 |
| 6-month | 427.4 (3.3) | 421.7 (3.3) | -1.1 (-10.6, 8.4) | 0.82 | 0.04 |
| 12-month | 435.3 (3.4) | 427.8 (3.3) | -0.7 (-9.0, 10.5) | 0.88 | 0.02 |
| *Time in bed (min)* |  |  |  |  |  |
| Baseline | 500.9 (3.2) | 493.7 (3.2) | .. | .. | .. |
| Post-session 2 | 483.7 (3.4) | 491.7 (3.4) | -15.3 (-25.0, -5.5) | 0.002 | 0.47 |
| Post-session 4 | 480.5 (3.6) | 490.2 (3.6) | -16.9 (-27.1, -6.7) | 0.001 | 0.52 |
| Post-intervention | 485.0 (3.4) | 491.0 (3.3) | -13.2 (-22.9, -3.6) | 0.007 | 0.41 |
| 6-month | 483.2 (3.5) | 488.3 (3.5) | -12.3 (-22.4, -2.2) | 0.02 | 0.38 |
| 12-month | 492.4 (3.6) | 494.3 (3.6) | -9.1 (-19.4, 1.2) | 0.09 | 0.28 |
| *Sleep efficiency, %* |  |  |  |  |  |
| Baseline | 80.4 (0.4) | 80.3 (0.4) | .. | .. | .. |
| Post-session 2 | 84.9 (0.5) | 83.5 (0.5) | 1.2 (-0.1, 2.5) | 0.07 | 0.29 |
| Post-session 4 | 87.7 (0.5) | 84.9 (0.5) | 2.7 (1.3, 4.0) | < 0.001 | 0.64 |
| Post-intervention | 89.0 (0.5) | 86.0 (0.5) | 2.8 (1.5, 4.1) | < 0.001 | 0.68 |
| 6-month | 88.7 (0.5) | 86.9 (0.5) | 1.6 (0.3, 3.0) | 0.02 | 0.43 |
| 12-month | 88.8 (0.5) | 87.0 (0.5) | 1.6 (0.2, 3.0) | 0.03 | 0.42 |
| *GAD-7* |  |  |  |  |  |
| Baseline | 7.7 (0.2) | 7.9 (0.2) | .. | .. | .. |
| Post-session 2 | 5.8 (0.2) | 6.3 (0.2) | -0.3 (-0.9, 0.3) | 0.40 | 0.14 |
| Post-session 4 | 4.6 (0.2) | 5.6 (0.2) | -0.8 (-1.4, -0.2) | 0.01 | 0.42 |
| Post-intervention | 4.0 (0.2) | 4.9 (0.2) | -0.7 (-1.3, -0.1) | 0.02 | 0.38 |
| 6-month | 4.3 (0.2) | 5.0 (0.2) | -0.6 (-1.2, 0.0) | 0.06 | 0.31 |
| 12-month | 4.5 (0.2) | 5.2 (0.2) | -0.5 (-1.1, 0.2) | 0.15 | 0.24 |
| *SSI-C^c^* |  |  |  |  |  |
| Baseline | 0.17 (0.02) | 0.15 (0.02) | .. | .. | .. |
| Post-intervention | 0.12 (0.02) | 0.13 (0.02) | -0.03 (-0.08, 0.02) | 0.23 | 0.17 |
| 6-month | 0.12 (0.02) | 0.11 (0.02) | -0.01 (-0.06, 0.04) | 0.81 | 0.04 |
| 12-month | 0.14 (0.02) | 0.12 (0.02) | -0.01 (-0.06, 0.04) | 0.77 | 0.05 |
| *MFI* |  |  |  |  |  |
| Baseline | 66.0 (0.8) | 64.8 (0.8) | .. | .. |  |
| Post-intervention | 53.7 (0.8) | 57.3 (0.8) | -4.8 (-7.0 to -2.6) | < 0.001 | 0.72 |
| 6-month | 52.3 (0.9) | 56.0 (0.9) | -4.9 (-7.1 to -2.6) | < 0.001 | 0.74 |
| 12-month | 53.8 (0.9) | 56.8 (0.9) | -4.2 (-6.5 to -1.9) | < 0.001 | 0.63 |

**Table G. Other prespecified outcomes with imputed missing data due to participants reaching study endpoint before final follow-up (continued)**

| *rMEQ* |  |  |  |  |  |
| --- | --- | --- | --- | --- | --- |
| Baseline | 12.3 (0.2) | 12.4 (0.2) | .. | .. | .. |
| Post-intervention | 13.7 (0.2) | 13.1 (0.2) | 0.7 (0.3 to 1.1) | 0.002 | 0.60 |
| 6-month | 13.6 (0.2) | 13.1 (0.2) | 0.6 (0.2 to 1.0) | 0.007 | 0.53 |
| 12-month | 13.6 (0.2) | 12.9 (0.2) | 0.7 (0.3 to 1.2) | 0.001 | 0.67 |
| *DBAS-16* |  |  |  |  |  |
| Baseline | 7.1 (0.1) | 7.1 (0.1) | .. | .. | .. |
| Post-intervention | 5.4 (0.1) | 6.6 (0.1) | -1.2 (-1.4 to -1.0) | < 0.001 | 1.84 |
| 6-month | 5.5 (0.1) | 6.3 (0.1) | -0.8 (-1.0 to -0.6) | < 0.001 | 1.27 |
| 12-month | 5.7 (0.1) | 6.4 (0.1) | -0.7 (-1.0 to -0.5) | < 0.001 | 1.13 |

Abbreviations: CBT-I, cognitive behavioral therapy for insomnia; DBAS-16, brief version of Dysfunctional Beliefs and Attitudes about Sleep; GAD-7, Generalized Anxiety Disorder 7-item; HE, health education; MFI, Multidimensional Fatigue Inventory; rMEQ, reduced Horne and Östberg Morningness−Eveningness Questionnaire; SSI-C, Scale for Suicide Ideation-Current.

^a^ All values are adjusted for sex and insomnia severity at baseline.

^b^ Cohen’s *d* is defined as the adjusted intervention effect divided by the square root of estimated sample variance derived from the linear mixed-effects model.

^c^ Data are log-transformed prior to analysis to achieve near-normal distribution.

**Fig A. Sleep-promoting medication use by intervention group**


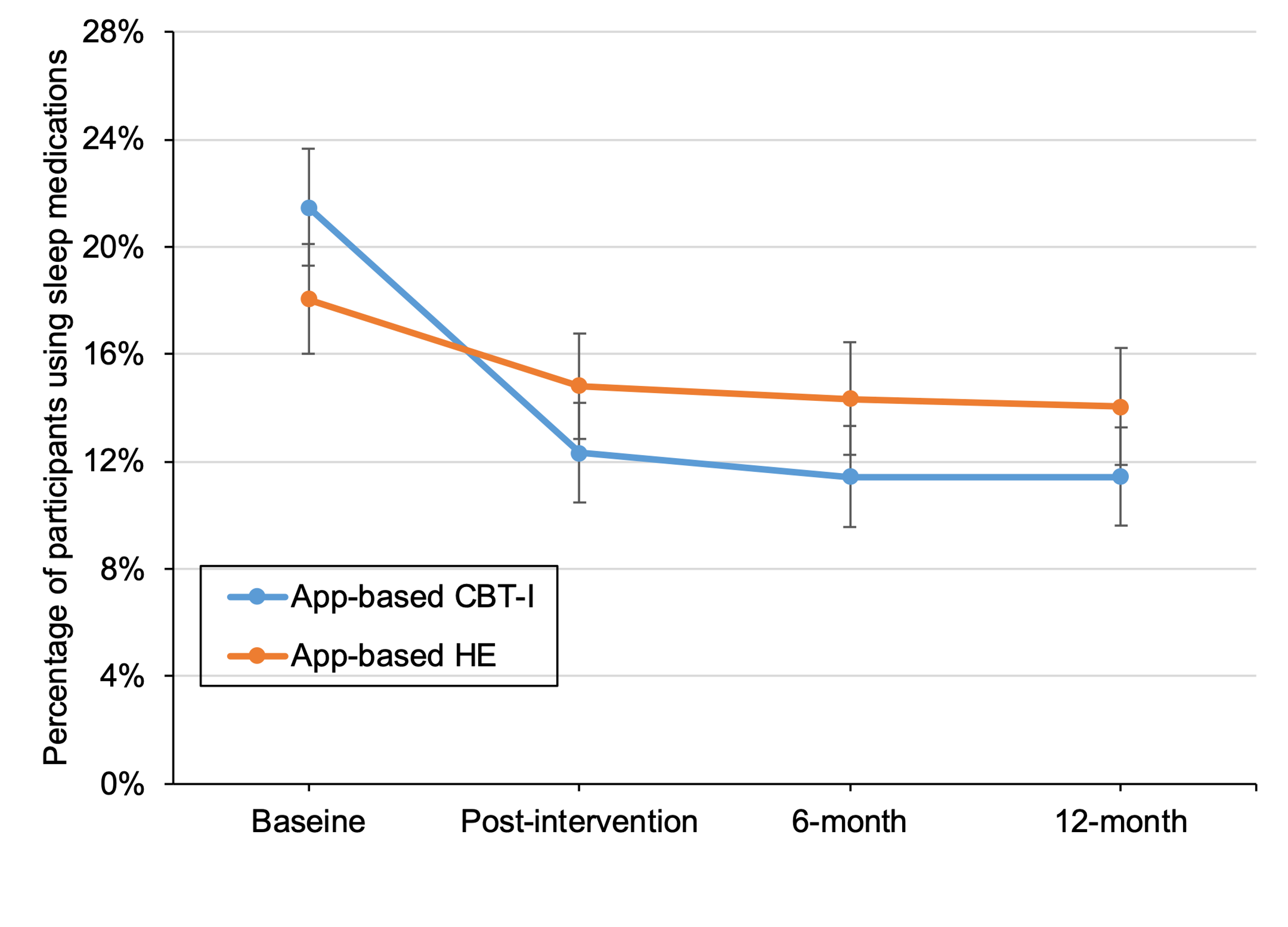


Error bars indicate standard errors. CBT-I, cognitive behavioral therapy for insomnia; HE, health education.

**Fig B. Risk of developing major depressive disorder by subgroups**


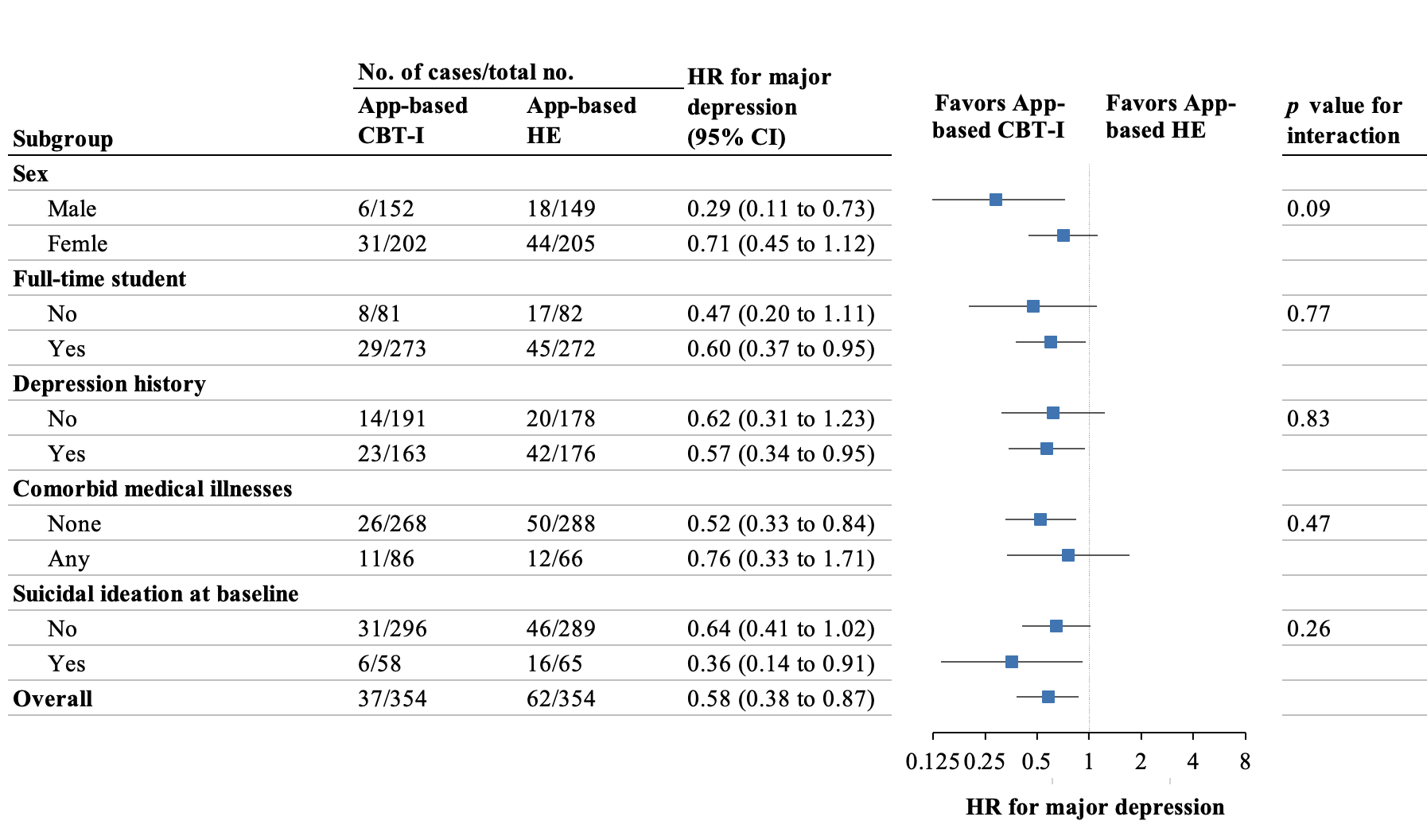


CBT-I, cognitive behavioral therapy for insomnia; HE, health education; HR, hazard ratio.

**Fig C. Risk of developing major depressive disorder by subgroups among participants with persistent insomnia**


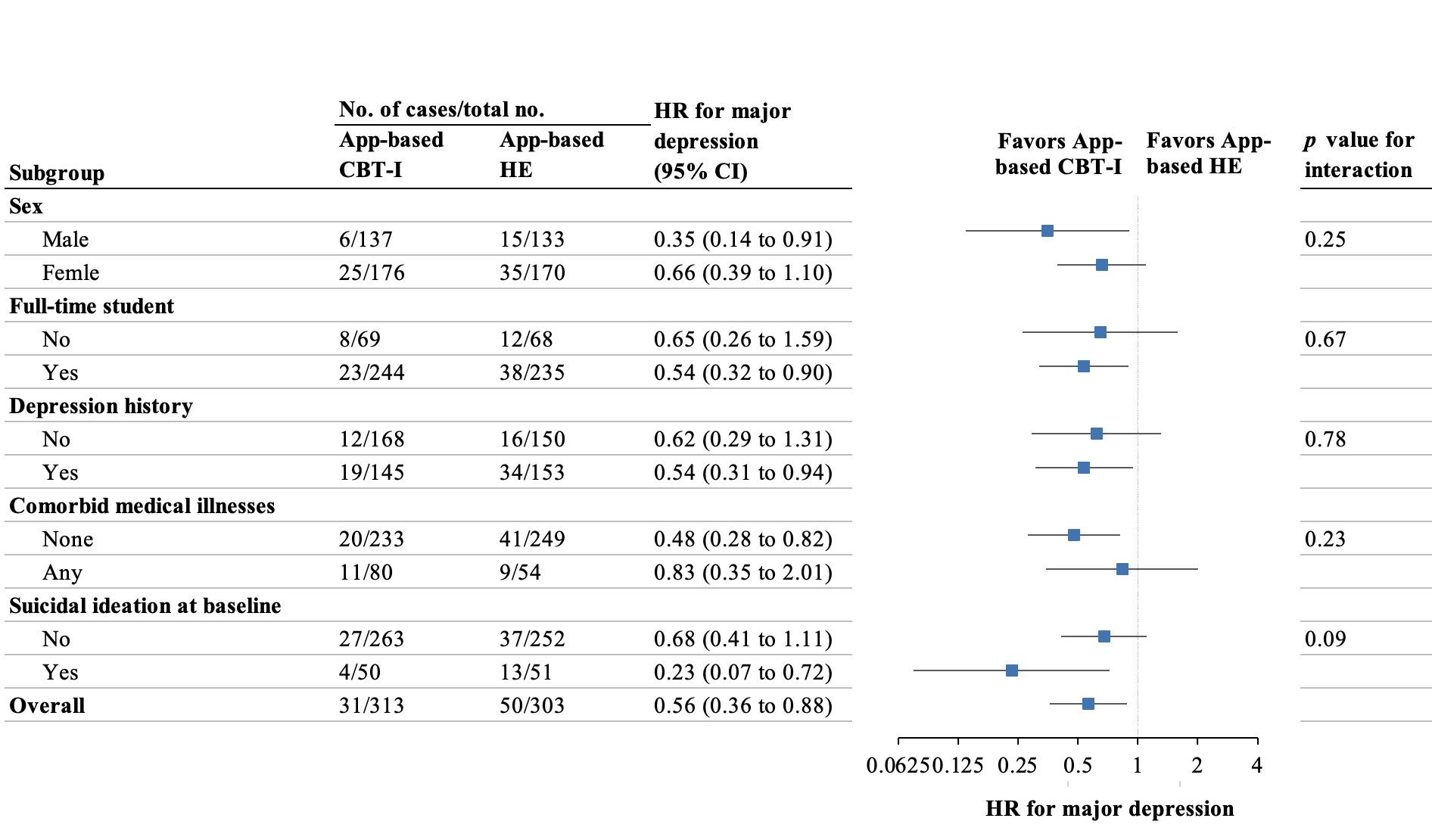


CBT-I, cognitive behavioral therapy for insomnia; HE, health education; HR, hazard ratio.

**Fig D. Remission rates of insomnia disorder by intervention group with imputed missing data due to participants reaching study endpoint before final follow-up**
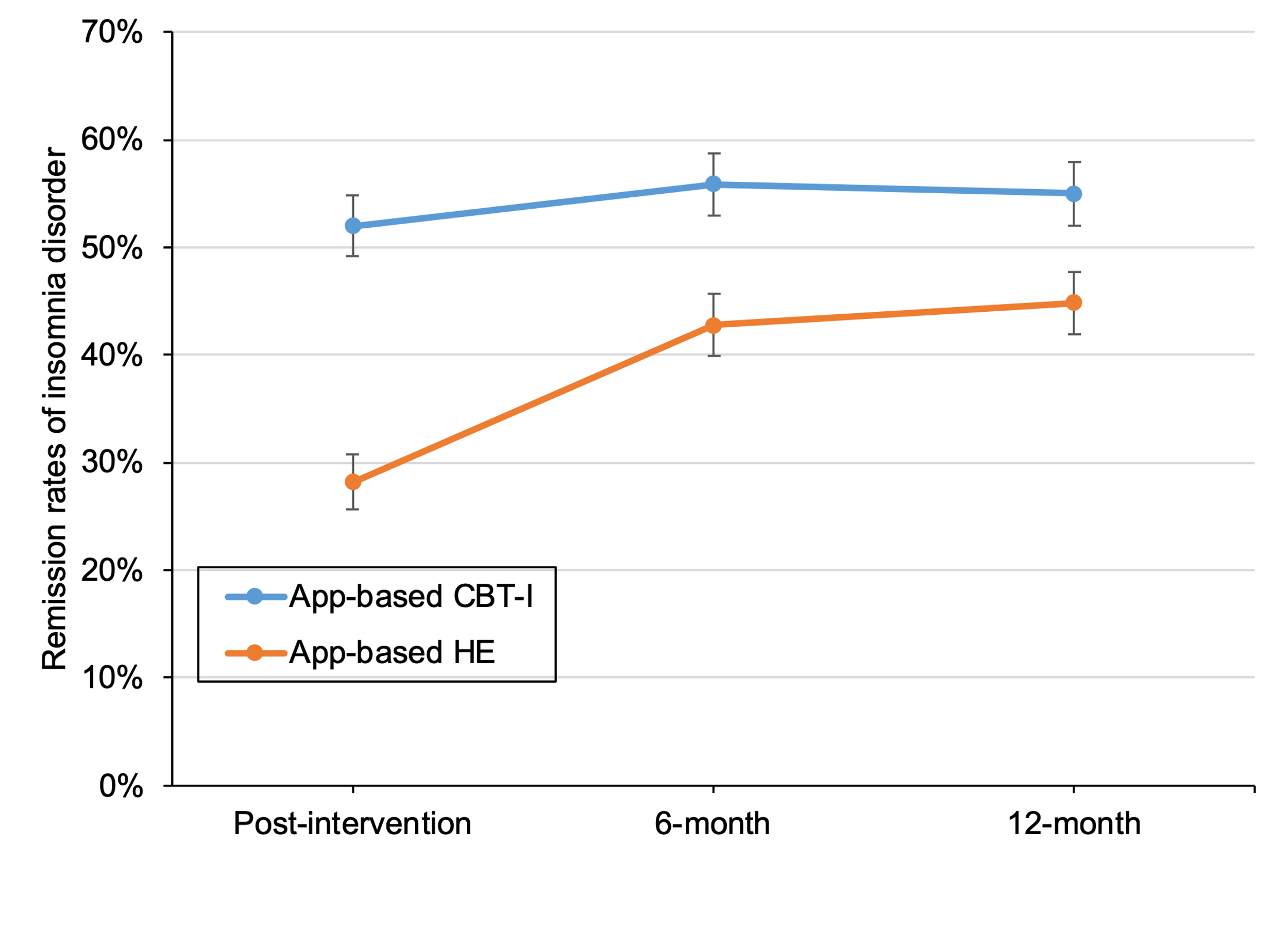


Error bars indicate standard errors. CBT-I, cognitive behavioral therapy for insomnia; HE, health education.

**Fig E. Comparison of secondary outcomes at each assessment**

**(A) Comparison of PHQ-9**


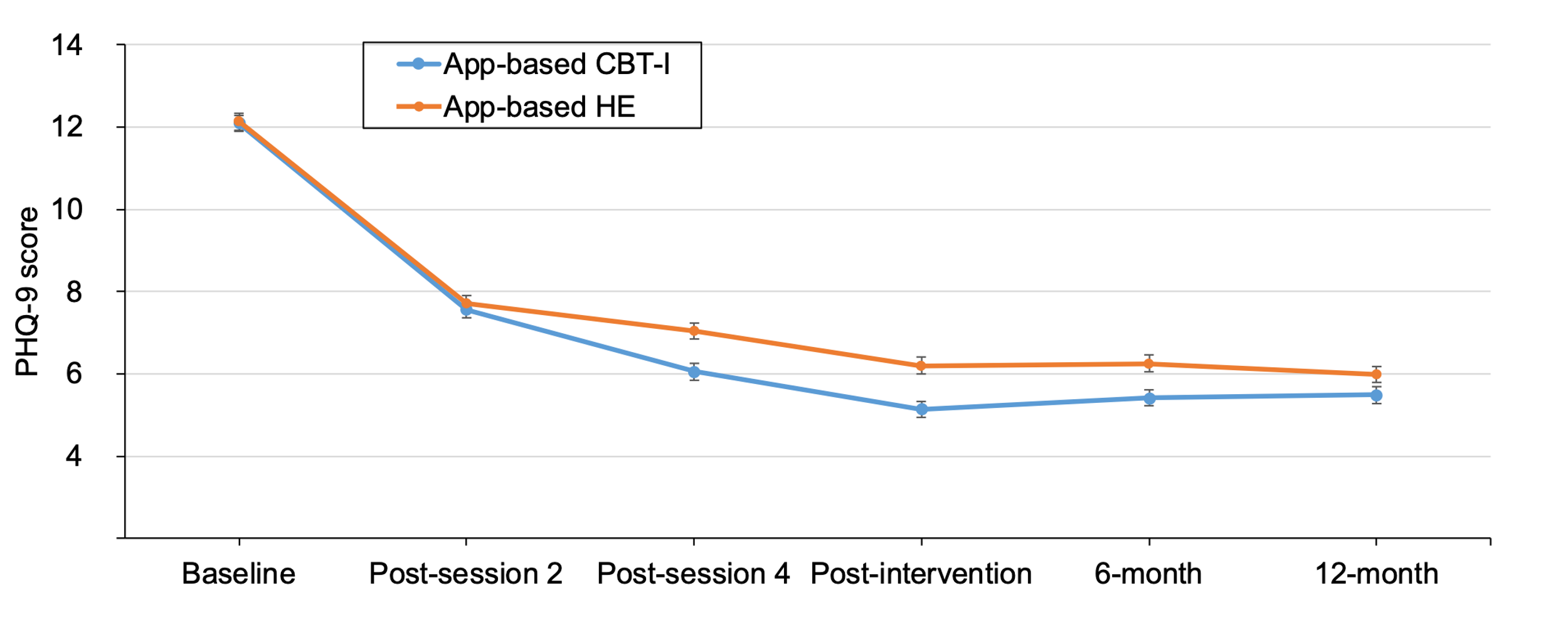


**(B) Comparison of PHQ-8**


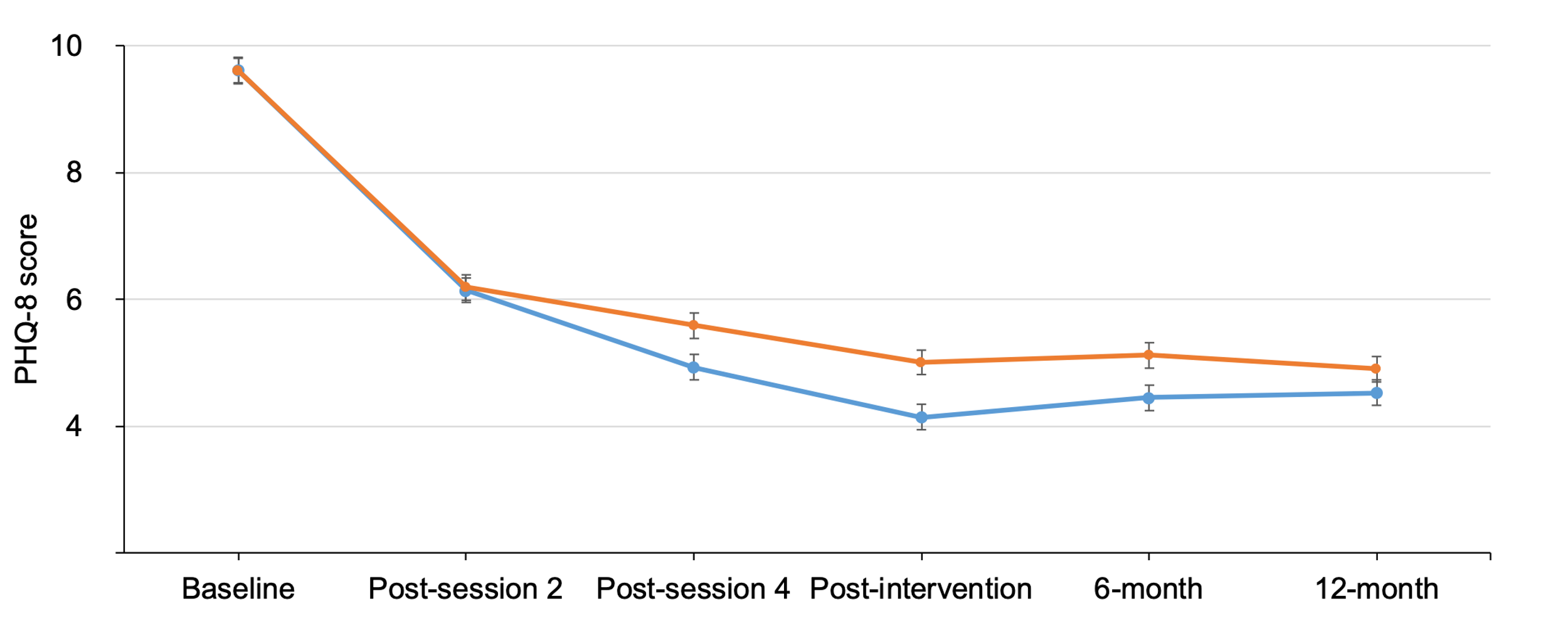


**(C) Comparison of ISI**


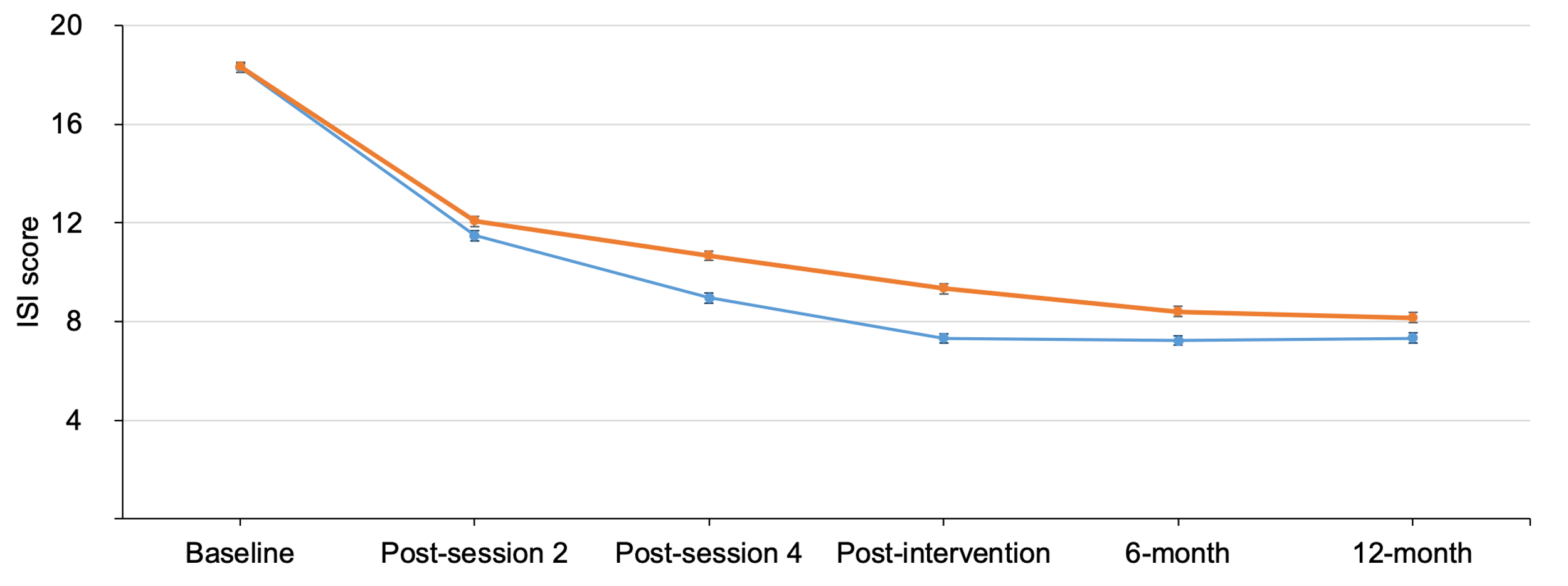


Error bars indicate standard errors. PHQ-8, PHQ-9 without sleep item. CBT-I, cognitive behavioral therapy for insomnia; HE, health education; ISI, Insomnia Severity Index; PHQ, Patient Health Questionnaire.

**Fig F. Comparison of other prespecified outcomes at each assessment^a^**

**(A) Comparison of sleep onset latency**

**(B) Comparison of wake after sleep onset**

**(C) Comparison of total sleep time**

**(D) Comparison of** **time in bed**

**(E) Comparison of sleep efficiency**


**(F) Comparison of GAD-7**


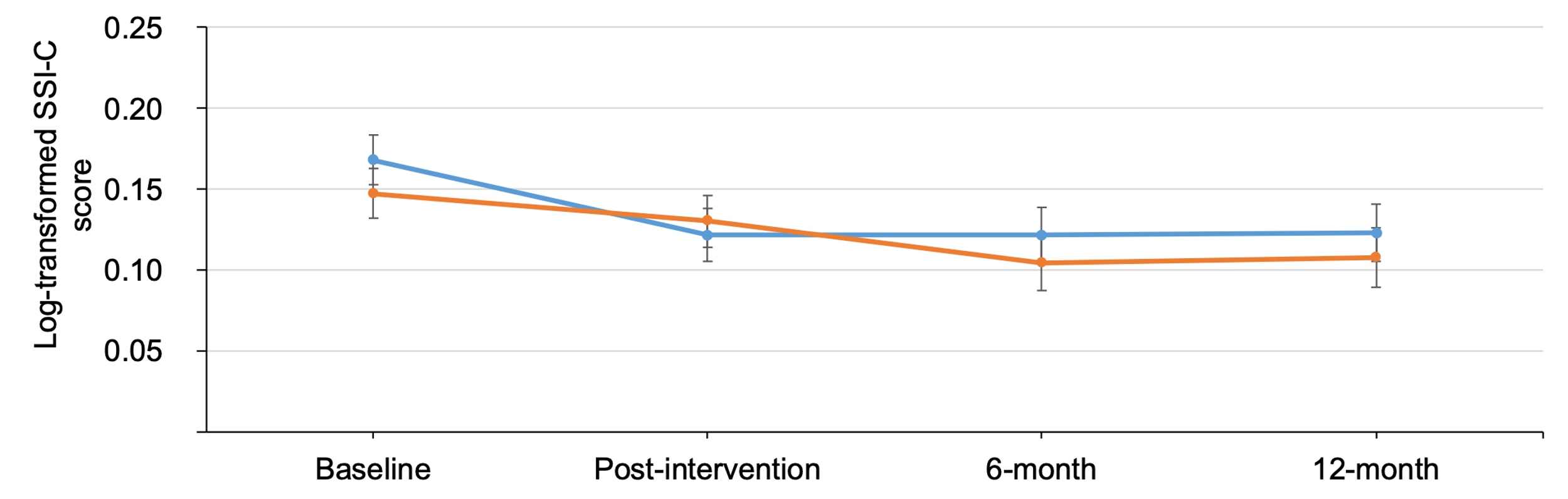
**(G) Comparison of SSI-C**

**(H) Comparison of MFI**


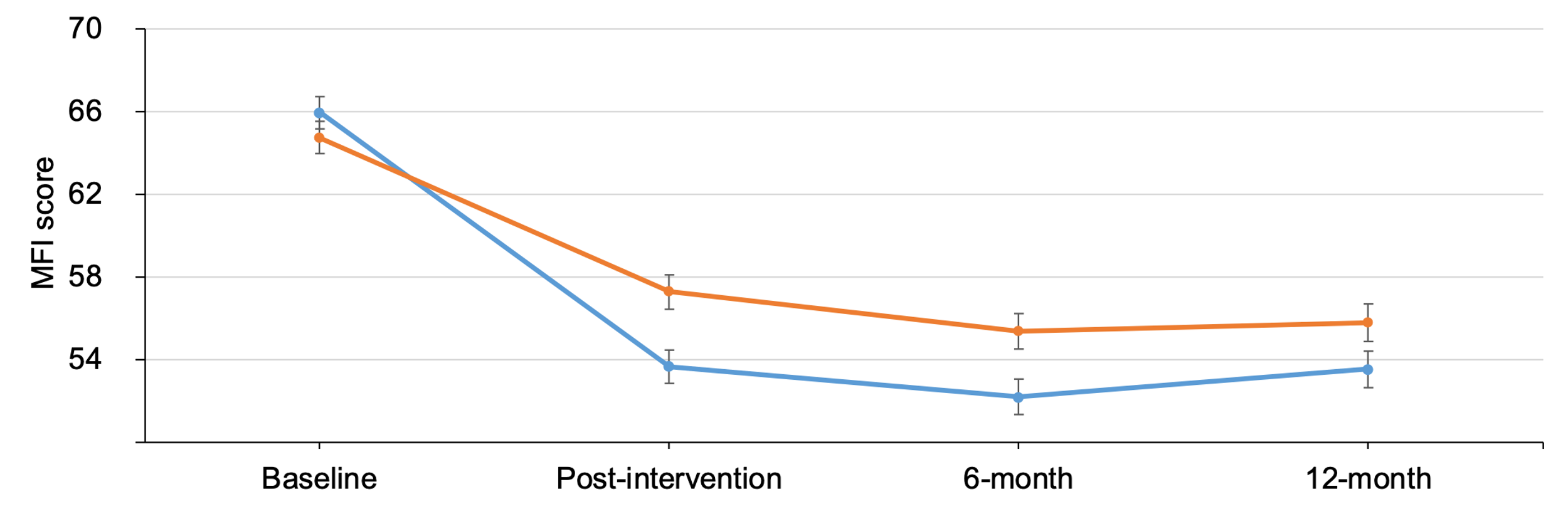


**(I) Comparison of rMEQ**

**(J) Comparison of DBAS-16**

Error bars indicate standard errors. CBT-I, cognitive behavioral therapy for insomnia; DBAS-16, brief version of Dysfunctional Beliefs and Attitudes about Sleep; GAD-7, Generalized Anxiety Disorder 7-item; HE, health education; MFI, Multidimensional Fatigue Inventory; rMEQ, reduced Horne and Östberg Morningness−Eveningness Questionnaire; SSI-C, Scale for Suicide Ideation-Current.
